# Development of a novel zebrafish xenograft model in *ache* mutants using liver cancer cell lines

**DOI:** 10.1038/s41598-018-19817-w

**Published:** 2018-01-25

**Authors:** M. Ender Avci, Ayse Gokce Keskus, Seniye Targen, M. Efe Isilak, Mehmet Ozturk, Rengul Cetin Atalay, Michelle M. Adams, Ozlen Konu

**Affiliations:** 1 Department of Molecular Biology and Genetics, Bilkent University, 06800 Ankara, Turkey; 2 Izmir International Biomedicine and Genome Institute (iBG-izmir), Dokuz Eylul University, 35340 Izmir, Turkey; 3 Medical Informatics Department, Graduate School of Informatics, Middle East Technical University, 06800 Ankara, Turkey; 4 Department of Psychology, Bilkent University, 06800 Ankara, Turkey; 5 Interdisciplinary Program in Neuroscience, Bilkent University, 06800 Ankara, Turkey; 6 UNAM-Institute of Materials Science and Nanotechnology, Bilkent University, 06800 Ankara, Turkey

## Abstract

Acetylcholinesterase (AChE), an enzyme responsible for degradation of acetylcholine, has been identified as a prognostic marker in liver cancer. Although *in vivo Ache* tumorigenicity assays in mouse are present, no established liver cancer xenograft model in zebrafish using an *ache* mutant background exists. Herein, we developed an embryonic zebrafish xenograft model using epithelial (Hep3B) and mesenchymal (SKHep1) liver cancer cell lines in wild-type and *ache*^*sb55*^ sibling mutant larvae after characterization of cholinesterase expression and activity in cell lines and zebrafish larvae. The comparison of fluorescent signal reflecting tumor size at 3-days post-injection (dpi) revealed an enhanced tumorigenic potential and a reduced migration capacity in cancer cells injected into homozygous *ache*^*sb55*^ mutants when compared with the wild-type. Increased tumor load was confirmed using an ALU based tumor DNA quantification method modified for use in genotyped xenotransplanted zebrafish embryos. Confocal microscopy using the Huh7 cells stably expressing GFP helped identify the distribution of tumor cells in larvae. Our results imply that acetylcholine accumulation in the microenvironment directly or indirectly supports tumor growth in liver cancer. Use of this model system for drug screening studies holds potential in discovering new cholinergic targets for treatment of liver cancers.

## Introduction

The most commonly occurring liver cancer is hepatocellular carcinoma (HCC), with over half a million new cases emerging yearly and a high death rate^1,2^. Hepatitis B and C virus infections are among the main factors underlying the development of HCC that is also associated with cirrhosis^3^. Exposure to aflatoxin B, chronic alcohol use, and other factors triggering cirrhosis also are etiologically important^4^. Moreover, liver cancer patients show differences in prognosis hence, it is important to discover and validate target genes affecting prognosis. Acetylcholinesterase (*ACHE*) is one such promising target^5^; changes in AChE amount has been associated with cancer prognosis or stage in different types of cancers including HCC^5–7^. AChE is responsible for enzymatic degradation of acetylcholine (ACh) and its absence or low activity causes high levels of systemic ACh^8^. ACh or the agonist nicotine binds to nicotinic ACh receptors fine-tuning cellular signal transduction^9^. As expected, there is an inverse correlation between AChE activity and ACh amount in liver cancer cell lines and in addition a decrease in AChE activity is associated positively with tumor size, multiplicity and TNM (tumor-node-metastasis) stage^5^. Moreover, in HCC patients with poor prognosis, there is a statistically significant decrease in the AChE amount^5^. Recombinant AChE treatment decreases cell proliferation in liver cancer cell lines and overexpressing AChE in a nude mice xenograft model decreases tumor size^5^. In light of these findings decreased AChE amount is associated with tumorigenesis and thus, studying its role in tumor microenvironment has potential implications in cancer diagnosis and therapy. However, currently there is no *in vivo* xenograft model for evaluating the connection between AChE and liver cancer development and progression in zebrafish.

Xenograft models for liver cancer have been developed in different organisms^10–12^. In mice, ectopic or orthotopic implantation of hepatoma cells from patient tissue or cultured cell lines into athymic (nude) or severe-combined immunodeficient (SCID) mice have been used for drug screening^13,14^. Use of multiple tumor cell lines has been proposed since there is a possibility of observing different tumor phenotypes^13,15,16^. Mouse model of xenotransplantation, however, presents challenges such as long tumor development periods and few numbers of cubs in a single born. Recently, the zebrafish has emerged as a model for tumor xenotransplantation studies^17–19^. Having hundreds of transparent offspring enabling ease in imaging and lack of early developmental adaptive immune system are the primary advantages of using the zebrafish embryos for transplantation. In addition, availability of mutant and transgenic strains with different genetic backgrounds might open new avenues for xenotransplantation^19–23^.

The zebrafish homolog of the human *ACHE* gene has been well characterized^24,25^. *ache*^*sb55*^ fish have a loss-of-function point mutation changing Ser226 residue to Asn in the catalytic triad of AChE active site resulting in a null phenotype^25^. Zebrafish *ache* homozygous mutants are initially motile but paralyzed at around 72 hours post-fertilization (hpf), whereas heterozygote mutants develop normally until adulthood. However, there is no established xenograft model in zebrafish using an *ache* mutant background in the literature.

Moreover, ALU based transplant DNA quantification methods have been used to quantify tumor load in mammalian xenotransplantation studies^26–29^. These methods provide high detection power and accuracy even when considerably lower proportion of human cells are found in host (<1e-4%) as well as with lower DNA amounts (0.1 pg)^28^. However, there is yet to be an ALU based quantification assay to be developed also in zebrafish.

In the present study, two representative liver cancer cell lines (Hep3B and SKHep1) with differential cholinesterase expression/activity were selected for transplantation. Next, we developed a novel model for comparison of tumorigenesis between phenotypically wild-type and *ache* mutant sibling embryos. Using microscopic imaging, we observed that *ache* mutant embryos developed larger tumors than wild-type siblings. Confocal microscopy enabled better visualization of tumor distribution by making use of an antibody against GPF that is stably expressed in Huh7 cells injected to zebrafish embryos. Next, we modified a mammalian ALU xenograft DNA quantification assay for use in single zebrafish embryos injected with cancer cells. Results showed that *ache* mutants contained higher numbers of tumor cells when compared with *ache* heterozygotes and homozygous wild-type embryos. Overall our findings indicate that in the absence of *ache*, excess systemic ACh in zebrafish larvae provides a permissive microenvironment for tumor development directly or indirectly. The effect we observed is relatively independent of the liver cancer cell cholinesterase expression but rather emphasizes differences in the host zebrafish *ache* expression. This is the first study of an effective xenograft model in zebrafish larvae using a mutant background for liver cancer cell xenotransplantation. This model holds potential for analyzing the tumorigenic capacity of cell lines and cells from primary tumors, and in combination with drug treatment experiments, offers a promising setup for preclinical trials.

## Results

### A novel xenograft model using liver cancer cells in *ache*^*sb55*^ mutant zebrafish

**Figure 1:**
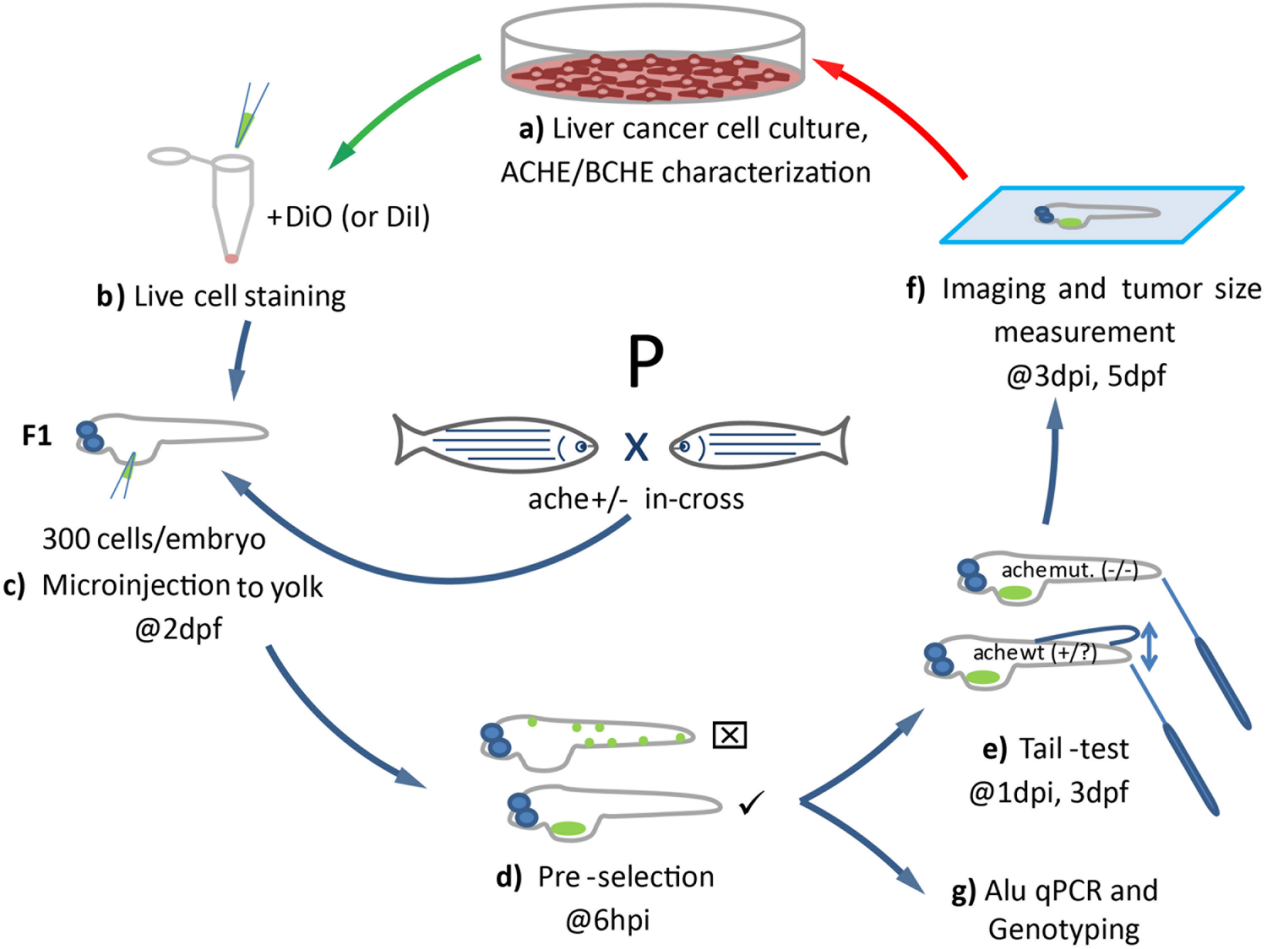
Schematic representation of the development of the liver cancer xenograft model in zebrafish *ache* mutants. (**a**) Liver cancer cells are grown in culture and characterized for *ACHE*/*BCHE* expression and activity. (**b**) Selected cell lines are harvested and stained with live dye DiO (or DiI for ALU quantification). (**c**) Embryos from *ache* heterozygous in-cross are injected with 300 cells into their yolk sac at 2 dpf. (**d**) At 6 hpi, embryos with positive signal are selected and embryos with signal from outside the injection site are not used. (**e**) After blind injection, mutant larvae are separated from +/? by a touch-evoked tail response test (tail-test). (**f**) All larvae are fixed and mounted before tumor size is measured and compared between mutant and wild-type larvae. (**g**) Alternatively, larvae fixed for ALU based xenograft quantification assay.

We developed an effective xenograft model using liver cancer cells in *ache* mutant background (Fig. 1). First, we characterized six different liver cancer cell lines in terms of their acetylcholinesterase/butyrylcholinesterase (*ACHE*/*BCHE*) expression and activity (Fig. 1a). Next, we selected for transplantation two different cell lines, namely Hep3B and SKHep1, respectively expressing *ACHE*/*BCHE* mRNA in low and high amounts. After staining with live dye DiO (or DiI for ALU quantification), these cells were injected into embryos at 2 days post fertilization (dpf) obtained from heterozygote *ache* fish in-cross (Fig. 1b,c). Following microinjection, at 6 hours post injection (hpi) embryos positive for tumor seeding were pre-selected, leaving embryos with fluorescent signal outside the injection site (yolk sac) out of further analysis (Fig. 1d). At 3 dpf, we performed touch-evoked tail response test (shortly “tail-test”) to separate *ache* wild-type and mutant embryos (Fig. 1e). Finally, embryos were fixed and mounted to take images, to measure tumor size and to compare wild-type and mutant siblings in terms of tumorigenesis and tumor cell migration (Fig. 1f). Alternatively, larvae were collected at the 3 dpi to extract DNA for ALU DNA quantification and zebrafish *ache* genotyping (Fig. 1g).

### Characterization of liver cancer cell lines based on *ACHE*/*BCHE* expression and activity

**Figure 2:**
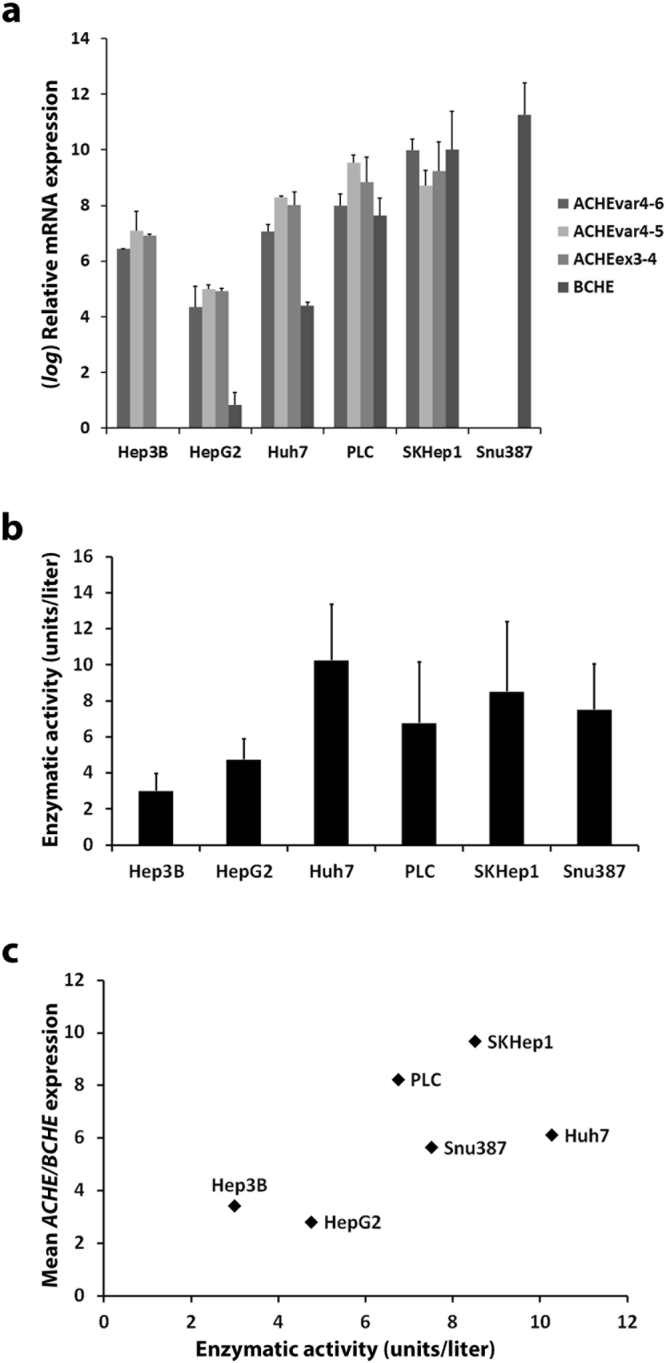
*ACHE*, *BCHE* expression and enzymatic activity profiling in selected liver cancer cell lines. (**a**) Human *ACHE* transcript variants *ACHE4-6*, *ACHE4-5* expression and another set of primers targeting *ACHE* exons no. 3 and 4 (*ACHEex3-4*) present in both variants were used for measuring relative *ACHE* expression by qPCR in the cell lines. Additionally, *BCHE* expression was measured by qPCR. Relative quantification was performed with respect to the cell lines with the lowest expression and *log* fold change represented. *TPT1* gene was taken as reference and ∆∆Ct method formula was used. (**b**) AChE enzymatic activity in HCC cell lines was measured using an Ellman method based commercial kit. (**c**) Mean *ACHE*/*BCHE* expression values and enzymatic activities were plotted for observation of correlation. Enzymatic activity numerical values were normalized by division to the highest value. All error bars represent the standard deviation.

For *in vitro* characterization of cell line *ACHE*/*BCHE* expression, we quantified *ACHE* and another functionally related gene, *BCHE* expressions using qPCR (Fig. 2a). In humans, there are two *ACHE* variants with either exon no.5 retained (*ACHEvar4–5*, NM_001302621.1) or exon no.6 retained (*ACHEvar4-6*, NM_001302622.1). We used another set of primers targeting *ACHE* exons no. 3 and 4 (*ACHEex3-4*) present in both variants. We found that all three amplicons exhibited correlated expression patterns allowing for estimation of an average *ACHE* transcript amount (Pearson’s correlation coefficients: rACHE4-6/ACHE4-5 = 0.96, *P* = 0.0025; rACHE4-5/ACHE3-4 = 0.99, *P* < 0.0001; rACHE4-6/ACHE3-4 = 0.98, *P* = 0.0004). *ACHE* transcripts were present in Hep3B, Huh7, PLC and SKHep1 cell lines. SKHep1 showed the highest relative expression whereas HepG2 had a lower and Snu387 virtually no *ACHE* expression (Fig. 2a). *BCHE*, on the other hand, was differentially expressed in PLC, SKHep1, Snu387 and to a lower extent in Huh7 but was almost totally absent in Hep3B and HepG2 (Fig. 2a). Next, we studied AChE/BChE enzymatic activity in our panel of liver cancer cell lines using a modified Ellman method based assay. In this assay Huh7, PLC, SKHep1 and Snu387 showed high enzymatic activities (Fig. 2b). SKHep1 had high levels of both *ACHE* and *BCHE* expression hence, expectedly it showed relatively higher enzymatic activity. Hep3B also, exhibited cholinesterase activity although not as highly as SKHep1. Our analyses revealed that total *ACHE*/*BCHE* mRNA expression exhibited a positive trend across averaged enzymatic activities (Fig. 2c, R = 0.65, *P* = 0.16). Additionally, we verified by Western blotting that the studied cancer cell lines exhibited AChE protein expression (Supplementary Figure 1).

### Characterization of *ache* expression and activity in zebrafish

**Figure 3:**
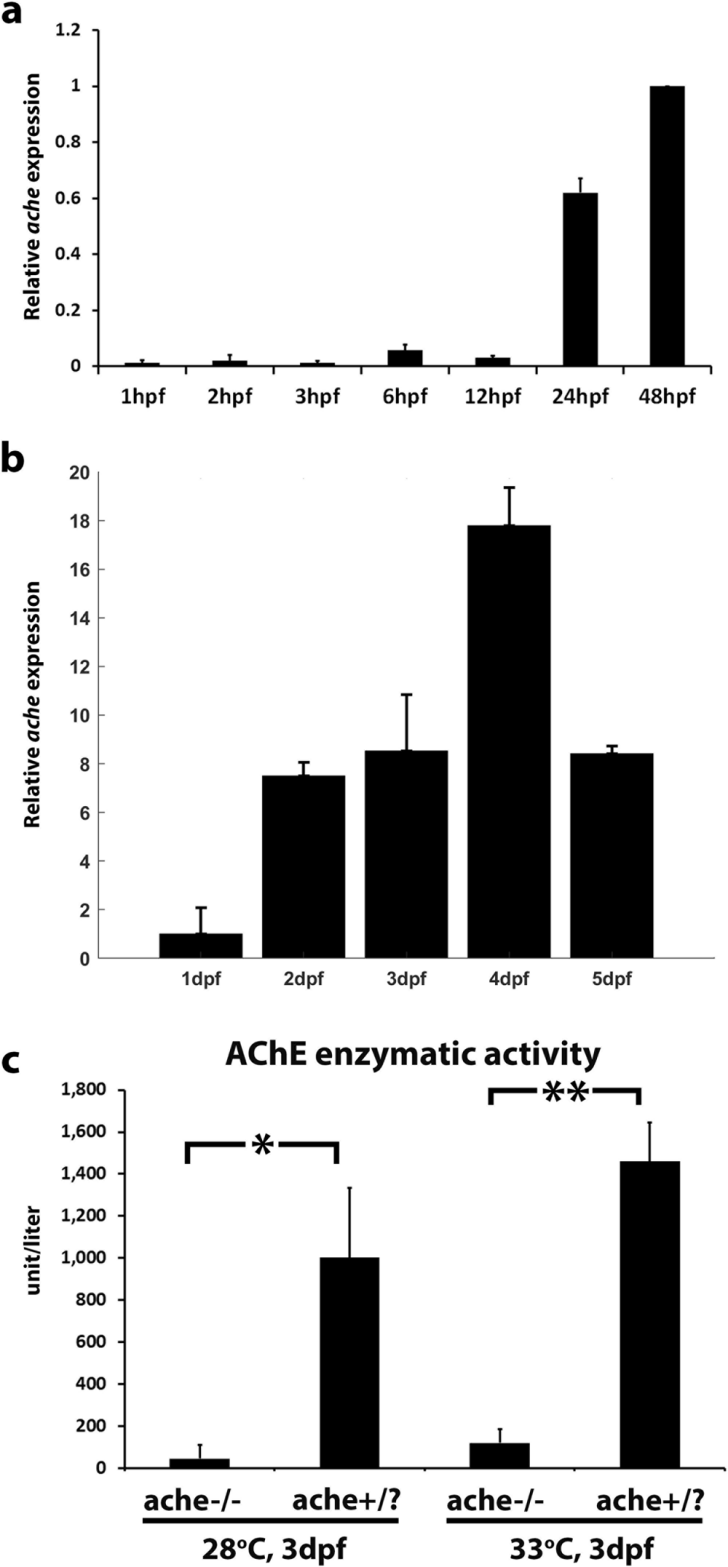
Temporal gene expression of zebrafish *ache* during embryonic and larval development. (**a**) Relative *ache* expression was quantified by qPCR at selected embryonic stages in AB line (N = 1 experiment, n = 20 embryos per group, standard deviation comes from technical replicates). qPCR reactions were run in duplicates and ∆∆Ct was calculated relative to 48 hpf values and normalized with *actb2* expression (**b**) Relative *ache* expression was quantified by qPCR at 1–5 dpf (N = 2 experiments, n = 20 embryo/larvae per group). qPCR reactions were run in duplicates and ∆∆Ct was calculated relative to 24 hpf values and normalized with *actb2* expression. (**c**) Normalized O.D. 412 values for groups of embryos. AChE enzymatic activity was almost absent in tail-test selected *ache*−/− mutant embryos when compared with wild-type *ache*+/? siblings at 28 °C (*P* = 0.034) or at 33°C (*P* = 0.003) (performed in triplicates, n = 8 embryos per group). Error bars show the standard deviation.

We performed qPCR experiments for determining the embryonic and larval expression levels of *ache* mRNA expression in zebrafish. We isolated RNA from 1, 2, 3, 6, 12, 24 and 48 hours post fertilization (hpf) embryos and verified *ache* expression quantitatively (Fig. 3a). We found higher zygotic *ache* expression at 24 and 48 hpf (also see Supplementary Figure 2a and b for RT-PCR and qPCR, respectively). We then synthesized anti-sense *ache in situ* probes and performed WISH using larvae at 24 hpf and 48 hpf (Supplementary Figure 2c,d). At 24 hpf *ache* expression was confined to myotome (Supplementary Figure 2c), whereas at 48 hpf it was restricted to the inner brain where ventrorostral and ventrocaudal clusters were located (Supplementary Figure 2d). These findings were in accord with *ache*’s established role in early neurogenesis^30^. We further studied the *ache* expression at later developmental stages in wild-type embryos up to 5 dpf using qPCR (Fig. 3b; N = 2 experiments with n = 20 embryos/group). This revealed that *ache* expression was present at all developmental time points studied, with the highest expression observed at 4 dpf.

We obtained *ache*^*sb55*^ founder fish from European Zebrafish Resource Center (EZRC) at 3 dpf and raised them to adulthood. For heterozygous parental fish identification, we performed touch-evoked escape response test (shortly “tail-test”) in embryos obtained from founder fish as described previously^25^. Zebrafish *ache*^*sb55*^ heterozygous (+/−) or wild-type (+/+) (together from here on designated as +/?) siblings were able to swim normally upon touching tails (Supplementary Figure 3, Supplementary Movie 1) while (−/−) homozygous mutant embryos were paralyzed at 72 hpf and were not able to contract their tail muscles due to high levels of ACh in the absence of AChE enzyme^25^ (Supplementary Figure 3, Supplementary Movie 2). Founder fish producing homozygous mutant embryos were selected and labeled as *ache*(+/−) heterozygous parents, and *ache*(−/−) homozygous mutant embryos and *ache*(+/?) siblings gathered from breeding of these fish were used further in xenotransplantation experiments (Fig. 1). To further verify the reliability of the tail-test and the identity of the heterozygous founder fish, we also measured AChE enzymatic activity in homozygous mutant (*ache*−/−) embryos identified by tail-test to compare with that of the siblings (*ache*+/?). As expected, we observed 22-fold higher AChE enzymatic activity in siblings when compared to *ache* mutant embryos at 28 °C (Fig. 3c). Since, after injection we kept the embryos at 33 °C for tumor development, we also checked AChE activity at this temperature and found a 12-fold higher activity in wild-type embryos (Fig. 3c). Enzymatic activity reflects only that of AChE since zebrafish has no *bche* gene^24^.

### Differential tumorigenic and metastatic capacity of *ache*^*sb55*^ mutants and wild-type siblings using Hep3B and SKHep1 cell xenotransplants

**Figure 4:**
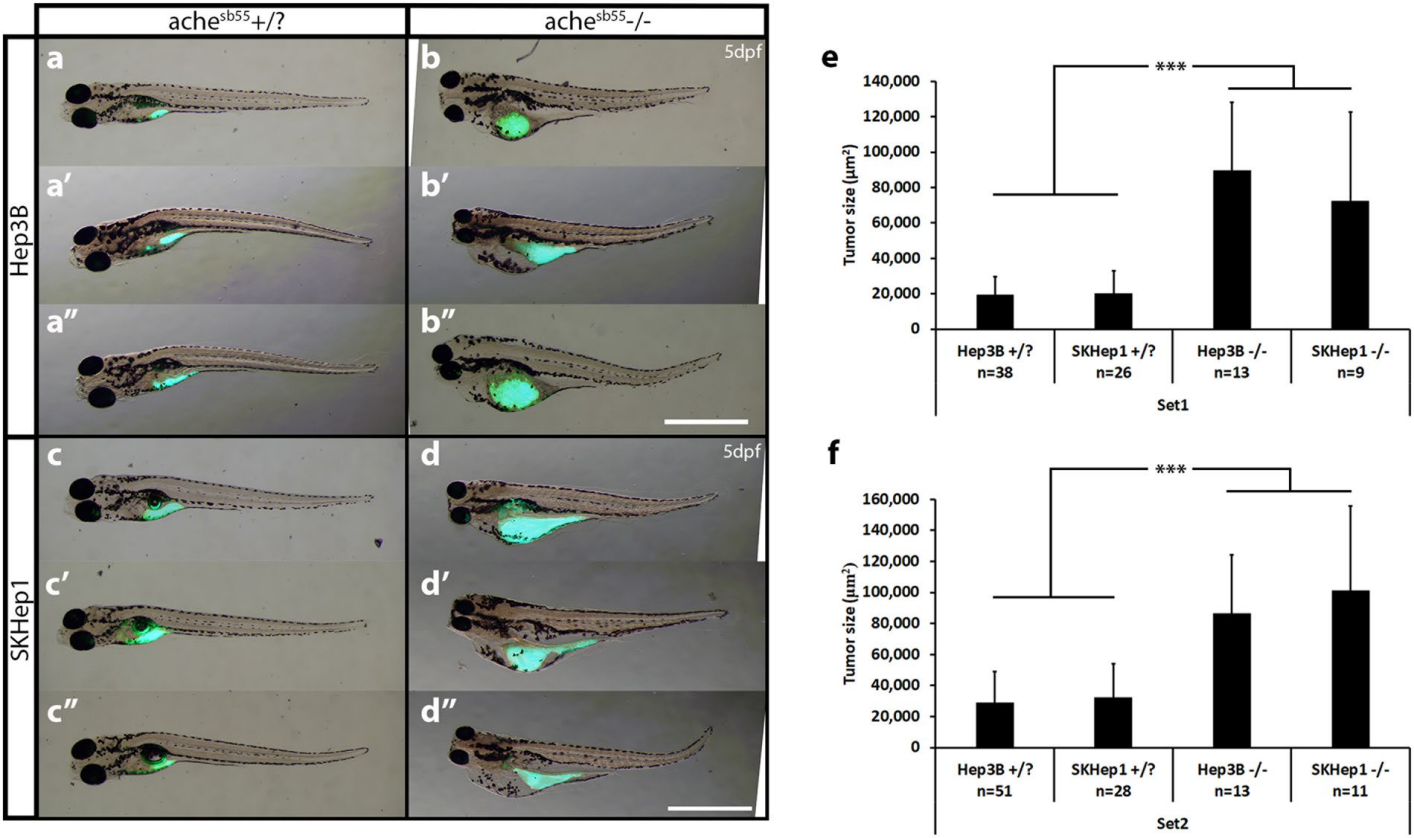
*ache* mutant embryos develop larger tumors with both Hep3B and SKHep1 human liver cancer cell xenotransplantation. At 2 dpf, embryos collected from *ache*+/− in-cross were injected with either Hep3B (**a**,**b**) or SKHep1 (**c**,**d**) cells. At 3 dpf, tail test was applied for phenotypically separating wild-type (**a**,**c**) and mutant embryos (**b**,**d**). At 3 dpi, larvae were fixed and imaged for analyzing tumor development (representative of Hep3B and SKHep1 injected groups, three different larvae are shown in a, a’, a”, **b**, b’, b” and **c**, c’, c”, **d**, d’, d”, respectively). Merged images from brightfield and GFP channels were shown. DiO labeled tumor masses can be clearly seen in green. Tumor sizes were measured in ImageJ using the fluorescent signal alone. In total 89 Hep3B injected *ache*+/?, 26 Hep3B injected *ache*−/−, 54 SKHep1 injected +/? and 20 SKHep1 injected −/− larvae were analyzed across two independent experiments. Results from each experimental set are separately graphed (**e**,**f**). Error bars represent the standard deviation. Bar = 1 mm.

**Figure 5:**
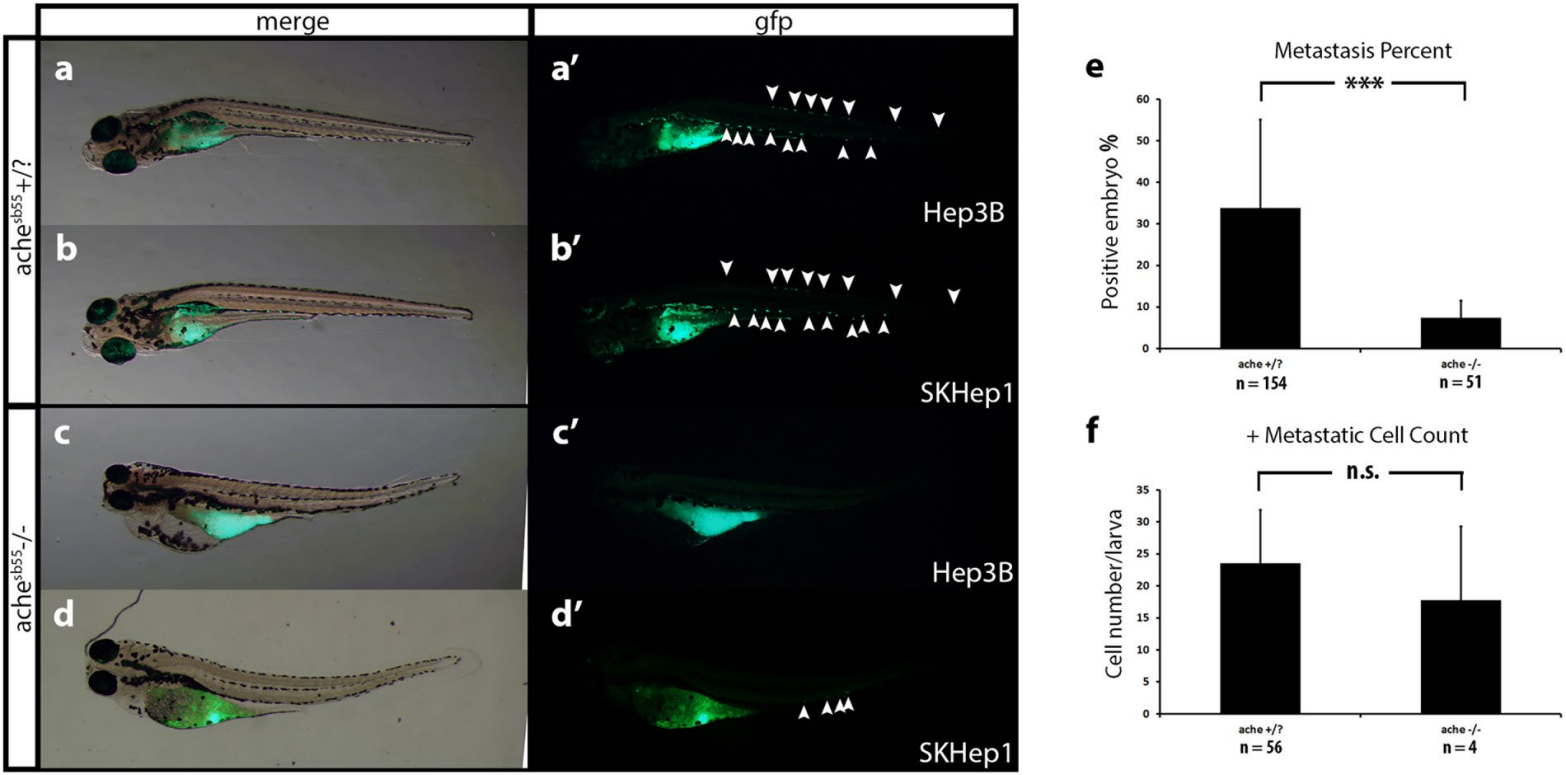
Comparison of metastasis in *ache* wild-type and mutant larvae. Representative metastatic larva images from Hep3B (**a**) and SKHep1 (**b**) injections into *ache*+/? (n = 154) larvae are shown. Green fluorescent dye DiO labeled migrated cells can be seen both in Hep3B (a’) and SKHep1 (b’) cell injections. Injection of Hep3B (**c**) and SKHep1 (**d**) into yolk sac of *ache*−/− (n = 51) larvae did not show a metastatic phenotype (c’,d’) (when there were more than 5 cells in tail region that embryo was counted as positive for metastasis, see (d’) which was counted as negative for metastasis). (**e**) Positive metastasis percent in *ache* wild-type and mutant groups were compared where there were significantly more metastatic larvae in *ache* wild-type group (*P* = 0.0002, chi-square test). (**f**) To see the effect of metastasis on local tumor size, tumor sizes of metastasis positive and negative groups were compared. Metastasis negative larvae (n = 129) showed a trend for larger tumors although non-significant (*P* = 0.1112, T-test) when compared to metastasis positive larvae (n = 60). n.s = not significant.

Based on qPCR expression analysis, Hep3B, known as a well-differentiated epithelial HCC cell line^31^, exhibited lower *ACHE*/*BCHE* expression/activity while a highly tumorigenic mesenchymal cell-line obtained from liver cancer adenoma^32^, SKHep1, showed high levels of *ACHE*/*BCHE* expression/activity. We developed a novel tumorigenicity model for these two cell lines comparatively, in the *ache* mutant embryos and healthy wild-type siblings in a microenvironment where *ache*, a marker for increased tumorigenicity and poor prognosis in human HCC, was depleted (Fig. 1). For this purpose, we injected Hep3B and SKHep1 (300 cells per embryo) to the center of yolk sacs of 2 dpf embryos obtained from *ache*^*sb55*/+^ heterozygote in-cross. At 3 dpf, we performed tail-test to separate mutant embryos from wild-type siblings and comparisons for tumorigenicity after cancer cell transplantation was done at 5 dpf using images taken by a fluorescent microscope (Fig. 4). When we compared the tumor size between *sb55* mutants and +/? siblings, we found that mutant embryos had significantly larger tumors as evidenced by the amount of fluorescent signal measured in two independent sets of experiments (Fig. 4a–d). In both Hep3B and SKHep1 injected embryos, in *ache* mutant background there were larger tumors (comparison between +/? and −/− in Fig. 4e,f). This difference in tumor size was independent of the cell lines used. In a Three-Way ANOVA analyzing the effect of phenotype, cell line and experimental sets on tumor size, we found that tumor size showed significant interaction with phenotype (*P* < 0.001). There was no significant interaction in between the other parameters such as cell line or experimental sets (*Pcell-line* = 0.757, *Pgenotype:cell-line* = 0.778, *Pgenotype:set* = 0.993, *Pcell-line:set* = 0.210, *Pgenotype:cell-line:set* = 0.113) yet experimental sets were different in terms of tumor size (*Pset* = 0.007). Injections to embryos with same genetic background did not differ in tumor size between experimental sets, whereas those with different backgrounds showed a significant difference in between phenotypes (ANOVA followed by Tukey comparisons: *P* < 0.001 for Hep3B+/? vs. Hep3B−/−, SKHep1+/? vs. SKHep1−/−, SKHep1−/− vs. Hep3B+/? and SKHep1+/? vs. Hep3B−/−). On the other hand, both Hep3B and SKHep1 injected mutants and +/? siblings exhibited comparable number of established tumors whereas migration was enhanced in +/? siblings apparent by a higher number of dispersed cells (Fig. 5a–e, Table 1; +/? = 37.8%, −/− = 6.6%; minimum 5 cells/embryo in the tail posterior to yolk sac taken as positively migrated^33^). Overall, this suggested that systemic ACh accumulation in the absence of *ache* function might form a permissive microenvironment for liver cancer cell proliferation and tumor formation.

**Table 1:** Tumorigenesis and Metastasis Percentages.

Cell line injected + ache genotype	Hep3B + ache−/− (n = 32)	Hep3B + ache+/? (n = 111)	SKHep1 + ache−/− (n = 28)	SKHep1 + ache+/? (n = 74)
No cells	6	12	5	9
Localized tumor	26	89	20	54
Invaded cells	0	28	4	28
Tumorigenicity %	81.3	80.2	71.4	73.0
Metastasis %	0.0	31.5	17.4	43.1

Liver cancer cell lines xenografted into *ache* mutant and siblings at 2 dpf, analyzed at 3 dpi.

**Figure 6:**
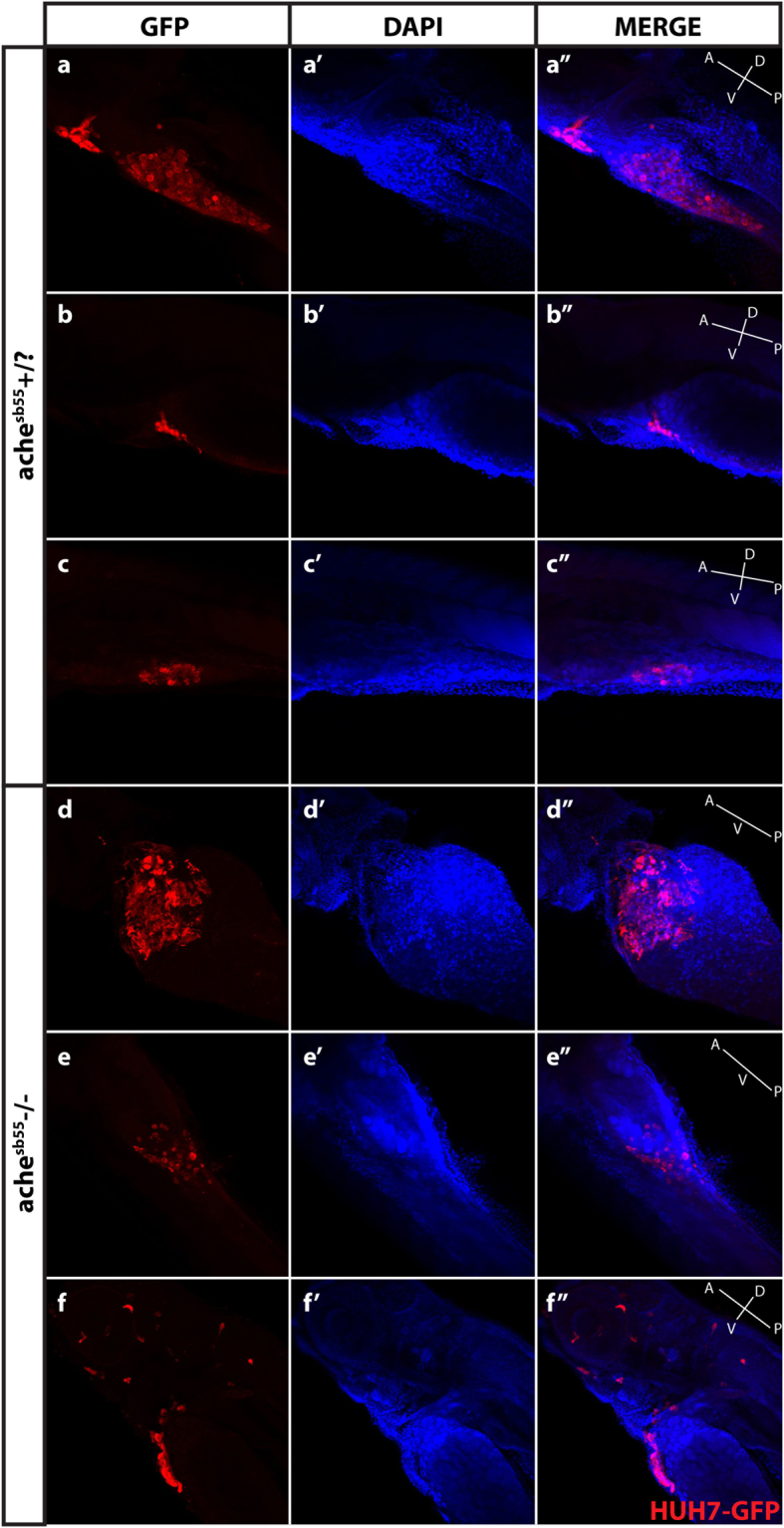
Optical sectioning and examination of Huh7-GFP xenografted larvae. Larvae (*ache*+/? and *ache*−/−) injected with Huh7-GFP cells were stained with GFP primary antibody and Cy3 tagged secondary antibody, and counterstained with DAPI and examined histologically by optical sectioning using a Zeiss LSM 880 confocal microscope. (**a**–**f**) At 20× magnification, z-stacks at 10 micron intervals were obtained and consecutive images were Z-projected to obtain these 2D images by maximum intensity projection in ImageJ. Tumor masses can be clearly seen in both images in red. (a’–f’) Same larvae were counterstained with DAPI for marking nuclei. (a”–f”) Merged images were presented. In the fish body schematic, the letters are abbreviations for *A*: anterior, *P*: posterior, *D*: dorsal, *V*: ventral.

Furthermore, we checked the histology of the xenografted tumors using confocal microscopy by optical sectioning where we used Huh7 HCC cells stably expressing GFP (G12 clone (pre-senescent) transfected with pEGFP-N2^34^). The histology of engrafted Huh7-GFP cells can be clearly seen in the yolk sac of representative *ache*+/? and *ache*−/− larvae at 5 dpf (3 dpi) stained with GFP antibodies (in red) (Fig. 6) where cell nuclei were counterstained with DAPI. GFP expressing cancer cells labeled with red could be seen settled in single or multiple foci in the yolk sac of the larva regardless of genotype of the fish (Fig. 6a–f). Furthermore, cancer cells could be clumped or dispersed in the larvae and at times metastasis to the brain and eye could be observed (Fig. 6f). However this method of stable fluorescent marker expression analysis is relatively time consuming and upon inclusion of other markers such as for proliferation or differentiation can be a promising alternative to the live cell staining with DiI/DiO, widely performed in other tumor settings^35–37^.

### AluYb8 DNA quantification by qPCR confirmed larger tumor volume in *ache* homozygotes

Prigent *et al*.^28^ used an AluYb8 based qPCR method to quantify human progenitor cells transplanted in rodent liver^28,38^ while the same set of primers were also used in pigs^29^. We have adopted a similar methodology to quantify human cancer cells in the *ache* mutant and wild-type zebrafish. In this model AluYb8 repeats were used to detect the human cell contribution in the mixture of human and host cells. We generated the standard curves in the presence of a constant amount of zebrafish DNA with 7-fold serial dilutions of human DNA (10 ng–0.01 pg). Consistent with the Prigent *et al*.^28^ the minimum detectable human DNA amount was found to be 0.1 pg. As expected there was a linear trend between the Ct values and the amount of human DNA in the human-zebrafish DNA mixture (Supplementary Figure 4; r = −0.998, *P* = 2.42e-7). In addition, we did not observe any cross reactivity of the AluYb8 primers with zebrafish DNA (Ct > 35). We did not observe any effect of amount of human DNA on the efficiency of the *ache* (wild-type and mutant specific) primers (See Supplementary Table 1).

DNA was extracted from 3 dpi whole larvae and directly used for qPCR. Primers previously defined^28,38^ were used for AluYb8 detection. We used *ache* (wild-type specific, S and mutant specific, N) primers for genotyping. DNA A_260_/A_280_ ratio on average was 1.89 ± 0.06 and 1.93 ± 0.1 for SKHep1 and Hep3B respectively while we used a value greater than 0.6 for the A_260_/A_230_ ratio and 30 ng/μl for concentration as thresholds for DNA quality. We examined the qPCR efficiency by comparing the *ache* Ct values and the log2 transformed DNA concentration levels of samples (Supplementary Figure 5). We obtained a significant negative relationship suggesting that concentration was a good predictor of *ache* (host) Ct values (r = −0.57 and *P* = 5.37e-7; r = −0.43 and *P* = 9.46e-4 for Hep3B and SKHep1, respectively). On the other hand, DNA concentration did not predict the AluYb8 (human DNA) content significantly (data not shown).

We also optimized the usage of the *ache* PCR primers for the genotyping of the heterozygote and homozygote zebrafish larvae. For the heterozygotes, we obtained detectable amplification with both primer sets. The correlation coefficient between the Ct values of each allele was highly significant (Supplementary Figure 6). For homozygous wild-type (*ache*+/+) larvae, the mean Ct difference between *ache* N - *ache* S primers was 9.85 (±1.07; SD) and 9.85 (±1.12; SD) while for homozygous mutant larvae (*ache*−/−) the mean Ct difference between *ache* N - *ache* S was −5.65 (±0.64; SD) and −5.7 (±0.8; SD), for SKHep1 and Hep3B, respectively. We considered the samples with an absolute Ct difference 1 and 4 cycles between primer sets as unclassified. With these primers, we successfully identified the genotype of the embryos; and the consistency of results showed the reliability of the genotyping (Supplementary Figure 6).

**Figure 7:**
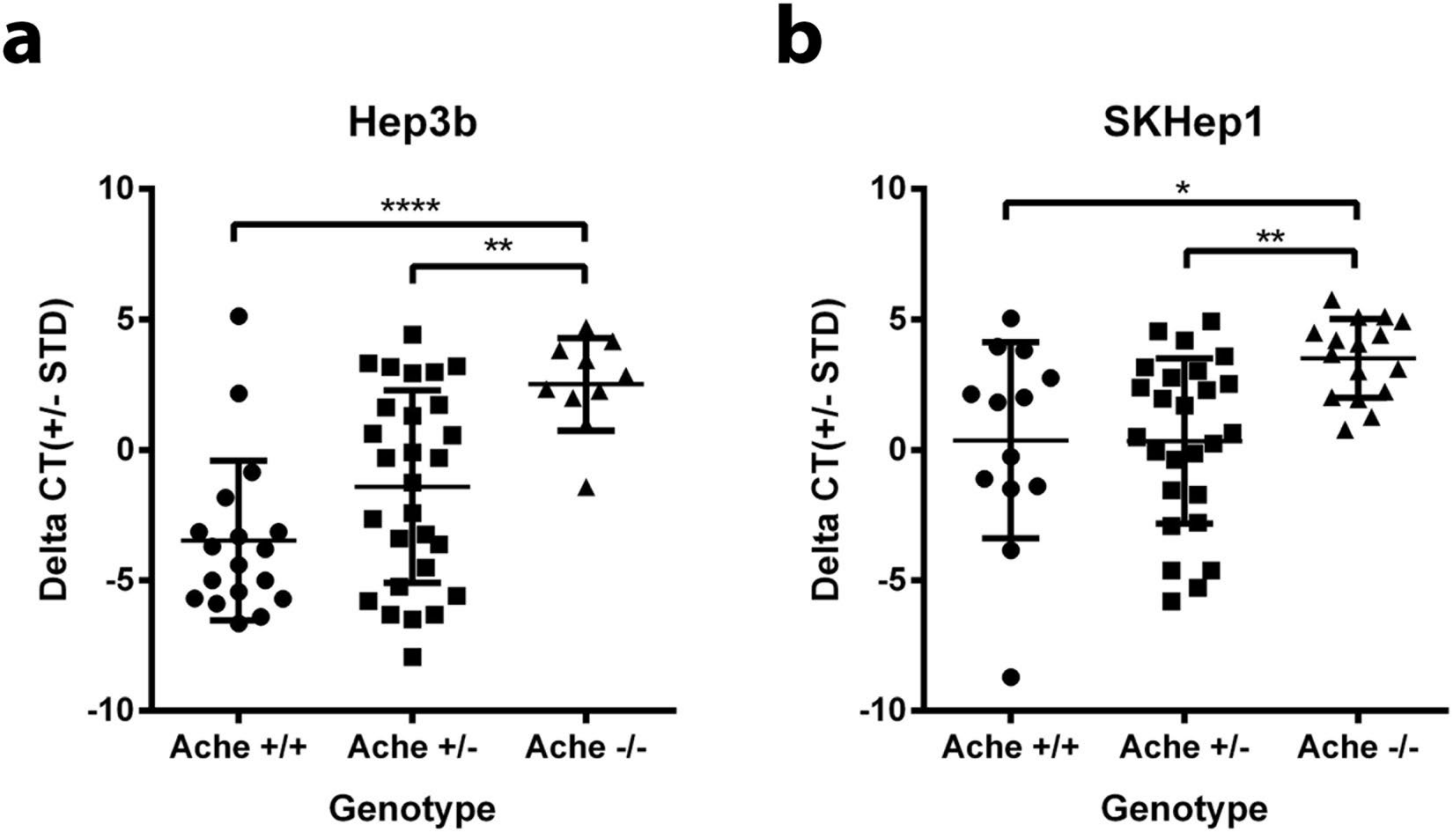
The comparison of the AluYb8 Ct values between genotypes using a qPCR assay. DNA was extracted from 3 dpi larvae injected with liver cancer cell lines (Hep3B and SKHep1) were amplified by qPCRs with using *ache* N, *ache* S, and AluYb8 primers (Supplementary Table 1). The Ct value difference (∆Ct) between *ache* N and *ache* S primers were used for the genotyping (Supplementary Figure 8). (**a**) For Hep3B (n = 18, 28 and 10 for *ache*+/+, *ache*+/− and *ache*−/−) normalized Ct values were significantly higher in *ache*−/− larvae in comparison to both *ache*+/− and *ache*+/+ larvae (*P* = 0.005 and 0.0001, respectively). (**b**) For SKHep1 (n = 13, 26 and 16 for *ache*+/+, *ache*+/− and *ache*−/−) normalized Ct values were significantly higher in *ache*−/− larvae in comparison to both *ache*+/− and *ache*+/+ (*P* = 0.004 and 0.017, respectively).

We next compared the ΔCt values of the AluYb8 primers between different genotypes. Consistently with the imaging results of tumor sizes, the ΔCt values were significantly high in *ache*+/? larvae compared to *ache*−/− (one tailed student’s T-test; *P* value = 3.11e-4, SKHep1; and 8.04e-4, Hep3B). When we compared all genotypes, we have observed that +/+ and +/− was not significantly different (*P* = 0.99 and *P* = 0.09 for SKHep1 and Hep3B, respectively) however, *ache*−/− was different from both *ache*+/+ and *ache*+/− (Fig. 7). We did not observe any significant interaction between the cell lines (Two-way ANOVA, *P* = 0.19).

## Discussion

Zebrafish and human liver carcinomas share similar molecular pathways^39^. Chemical treatment of zebrafish results in liver pathologies like steatohepatitis and hepatocellular carcinoma^40,41^. Recently, inducible oncogene-dependent transgenic models of HCC have been developed in zebrafish^12,42,43^. However, obtaining founder fish and growing F1 generation can be tedious and time-consuming. Thus, rapid and amenable procedures for high-throughput drug screening setups in mutant/transgenic zebrafish for treatment of liver cancer is an open avenue. In the present study, we developed a novel zebrafish xenotransplantation model using liver cancer cells with differing molecular and cell behavioral characteristics in *ache* mutant embryos in comparison with their phenotypically wild-type siblings.

Liver cancers are heterogeneous, hence cultured cell lines can provide opportunities to test functional consequences of differing molecular characteristics. For example liver cancer cell lines show different phenotypes in terms of liver marker expression like *AFP* and/or their epithelial or mesenchymal characteristics which need to be tested *in vivo* for their tumor forming and metastatic potentials^44,45^. In this study, using liver cancer cell lines expressing different amounts of *ACHE*/*BCHE* and epithelial or mesenchymal characters, we established a novel xenograft model in wild-type and *ache* mutant zebrafish larvae at 3 dpi. Previous studies have shown that *ache* in zebrafish is zygotically transcribed starting between 12–24 hpf ^24,46^ and enzymatically active. In addition, we have confirmed this using qPCR and *in situ* experiments and further extended the expression profile investigation to 5 dpf, establishing *ache* expression also in larval zebrafish.

We also showed that expressions of *ACHE* splice variants were highly correlated with each other in liver cell lines as a continuum of the findings of Zhao and colleagues^47^. Our findings suggested that *ache* mutants exhibited larger tumor volumes in comparison with healthy siblings regardless of the cell line used although they showed differential cholinesterase (*ACHE*/*BCHE*) expression.

*ACHE* expression has been shown to be downregulated in human HCCs^5^ and lower *Ache* levels have been implicated also, in cirrhosis induction in a rat model^48^. Parallel to these findings, we showed that presence of *ache*^*sb55*^ mutation in the host enabled enlarged tumor growth after xenotransplantation, suggesting that dysregulation of *ACHE* expression can be an important factor in liver cancer. ACh and AChE levels have also been associated with several other carcinomas like colon, prostate and breast^6,7,49^. Therefore, our *ache* xenograft model can further be tested using other human epithelial cancer cell lines to generalize our findings for liver cancer cells.

The strikingly larger tumor size in *ache* mutants in comparison to that in *ache* heterozygotes and/or wild-type embryos indicates that increased ACh levels in the host environment potentially leads to enhanced tumor size. ACh has been reported to stimulate colon and lung cancer cells in an autocrine manner^50,51^. Here, we provide evidence that systemic ACh accumulation in the absence of *ache* activity in host zebrafish, may result in enhanced liver cancer cell proliferation and tumor growth in this transplantation model using imaging and Alu-based DNA quantification assays. This observation could indeed be independent of *ACHE* expression profile in these cells. Another possibility is that the baseline activity of ACHE/BCHE protein in Hep3B might be sufficient so it performs similarly to SKHep1 with a higher ACHE/BCHE activity. The fact that heterozygote zebrafish *ache*+/− mutants do not exhibit paralysis supports this latter hypothesis. Rescue experiments performed with co-injection of different levels of *ache* mRNA might further help clarify whether the effects of excessive acetylcholine can be remedied in a dose-dependent manner by increased ache activity in mutant embryos. Future studies also should test the tumor size differences in transplantation of isogenic cell lines in which *ACHE* mRNA is knocked down or over-expressed within the same background. In addition, RNA or tissue from xenotransplanted tumors can be tested for changes in the expression level of known tumor markers.

Metastasis might partially contribute to observed tumor size difference between +/? and −/− larvae due to potentially larger tumor bulk in the yolk of metastasis-negative larvae (Fig. 5f, not significant). Alternatively, but not mutually exclusively, secondary factors, e.g., increased inflammation and edema might lead to tumors growing faster. Potentially the yolk area in which the tumors can grow might exhibit differences between the *ache* mutants and wild- type/heterozygote population resulting in easier growth due to larger size and/or differential levels of nutrients. Our comparative analysis of yolk sac size indicated that when not injected with tumor cells, mutant embryos have 11 to 25% larger yolk sac sizes compared to siblings at 3 and 5 dpf, respectively (Supplementary Figure 7). Given that when injected with cancer cells the resulting tumors are up to 3 folds larger in mutant embryos, the observed yolk sac size difference could not solely explain the enlarged tumor size. In addition, the body length of mutant larvae was decreased (about 0.5 mm at 5 dpf) in comparison with *ache* wild-type larvae, indicating a general developmental delay (Supplementary Figure 8). Furthermore, we observed an increase in dispersion of injected cells to tail in wild-type embryos in comparison with mutants. One explanation for the presence of lower number of dispersed cancer cells in *ache* mutants could be faulty circulation since inhibition of ache activity by organophosphate inhibitors has been shown to result in cardiac edema and decreased blood flow although the vasculature developed normally^52^. The positive green signal coming from DiO labeled dispersed cells mostly coincided with a signal obtained from the red channel potentially due to autofluorescence of iridophores^53^. Hence, it is possible that the dispersed cells use a similar route where iridophores migrate to reach their final physiological target locations and/or the red signal has bled through from the green. Xenotransplantation of GFP-labeled Huh7 cells in the +/? and −/− *ache* siblings was a first step taken in the present study for better visualization of the xenotransplants of liver cancer cells through confocal microscopy. Future studies should include optimization of multi-color staining as well as use of *ache* mutant hosts expressing fli-GFP for better tracking of the cells.

Human tumor cells originating from breast, adenocarcinoma, melanoma, pancreas, colorectum, glioma, ovary, prostate, and leukemia have been successfully xenotransplanted into zebrafish host previously (for a review see Konantz *et al*.^54^). Although there are different sites for injection (e.g. duct of cuvier, perivitelline or peritoneal cavities), the yolk is the most widely used location for transplantation. The number of injected cells ranges from as low as 40 up to 2000 cells. Recently, cancer stem cells from the HepG2 liver cancer cell line has been shown to form tumors and tail metastasis in an adult zebrafish xenograft model^55^. In a related study, HCC xenografts in zebrafish embryos were produced using Bel-7402 hepatoma cell line; and the effect of ADAM15 recombinant human disintegrin domain (rhddADAM15) was analyzed on tumor growth and metastasis^56^. In our study, we managed to implant tumors from human liver cancer cell lines in zebrafish yolk sac within a time frame as short as 5 dpf. Tumor sizes were estimated using 2D measurements commonly used in the literature^54,56,57^. Since the model uses zebrafish embryos, it is possible to screen drugs in a 96 well or even 384-well format because of the smaller body size and transparency of the embryos. We have the advantage of using human cells that more specifically reveal the molecular characteristics of clinical cases; and it would also be possible to use primary HCC cells, e.g. patient-derived xenografts^58^, as well as murine tumor cell lines or healthy hepatocytes in this *in vivo* setup. These above-mentioned arguments pinpoint the advantages of drug screening for liver cancer in the zebrafish *ache* model. Indeed in a similar recent study, the dynamics of a novel HCC cell line xenografted in zebrafish larvae was described demonstrating neo-angiogenesis and potential of a proteasome inhibitory drug to decrease tumor size and cell proliferation^57^.

In the present study, we also adopted a primate specific AluYb8-based quantification method, previously used in rodents and pigs, for detecting Hep3B and SKHep1 cell line DNA in the zebrafish xenograft model. To the best of our knowledge this is the first time to apply such methodology in zebrafish. Imaging and probe based assays provide semi-quantitative and/or highly variable data. However, a qPCR based model enables to detect and compare the results from single genotyped embryos. The variability in DNA quality and quantity affects the qPCR results but this can be tested and normalized using appropriate controls. For this study, we used quality control thresholds to eliminate low DNA quality samples and performed genotyping for *ache* in the same assay; thus, this allowed comparison of homozygote mutants against both the heterozygotes and homozygous wild-type embryos. Our findings suggested that the observed increase in tumor size was unique to homozygous mutants and no significant dose effect was apparent for the cell lines studied. We used the ΔCt measurements (human cell DNA normalized with zebrafish *ache* DNA) in our model to reduce the variability in the DNA concentration for estimating human DNA concentration. In future studies, different DNA extraction protocols might be tested to increase consistency of DNA concentration. In our model, we used DNA mixes from human cancer cells and zebrafish embryos for the standard curve generation. In the literature, it was reported that DNA mix based standard curves tended to underestimate the cell number in the rodent host^28,59^. Cell mixes may provide a more refined model for determination of the number of cells in the future.

In conclusion, we have developed a novel xenograft model in zebrafish embryos using a mutant background, i.e., the *ache*^*sb55*^ mutation, for human liver cancer cells. In *ache* mutants, the absence of host ache enzymatic activity directly or indirectly resulted in enlarged tumor size, increased tumor load, and in part decreased metastasis to tail region. The contribution of the yolk environment, vasculature, edema, and possible inflammation should be pursued and better characterized. Moreover, our mutant xenograft model and the AluYb8-based quantification method hold great potential to follow effects of cholinergic as well as known (e.g. Sorafenib) and novel drugs in an *in vivo* setup as a promising screening platform.

## Methods

### Ethics statement

The experimental protocols in this study were approved by Bilkent University Laboratory Animals Local Ethics Committee (Protocol no: 176, Decree no: 2013/48). All the applied methods were performed in accordance with the relevant guidelines and regulations.

### Animal care and handling

Zebrafish *ache*^*sb55*^ mutant and AB lines (obtained from European Zebrafish Research Center (EZRC)) were raised and staged according to the standard protocols^60^. The adult fish were maintained at 28 °C in aquaria with 14 hours of day and 10 hours of night cycles (ZebTEC, Italy). Embryos were kept in E3 medium at 28 °C until they reached the desired embryonic stage.

### DNA extraction

We used single 5 dpf embryos for the DNA extraction. Embryos were incubated in DNA extraction buffer (100 mM Tris-Cl, 10 mM EDTA, 200 mM NaCl and 0.5% SDS) and proteinase K (200 ug/ml). Isopropanol and 70% EtOH were used for the DNA precipitation and wash^61^.

### cDNA synthesis and quantitative real-time PCR

Total RNA from liver cancer cell lines and zebrafish embryos/larvae were isolated using Trizol reagent according to manufacturer’s instructions (Invitrogen, USA). cDNA was produced using oligodT primers from 1μg of total RNA with RevertAid First Strand cDNA Synthesis Kit (Thermo Fisher Scientific, USA).

PCR primers were designed in Primer3 software for gene specific RT-PCR experiments (Supplementary Table 1)^62^. Quantitative real-time PCR was performed as described previously using 2 × SYBR green mix (Roche, Switzerland) for human *ACHE* variants, *BCHE, AluYb8* and zebrafish *ache*^44^. Delta-delta Ct (∆∆Ct) method was applied for relative quantification using human *TPT1* and zebrafish *actb2* or *elfa* as reference genes^63^.

Delta Ct method was used for the relative quantification of the AluYb8 with using zebrafish *ache* gene as a reference. Due to the copy number difference between homozygous and heterozygous DNA, a value of 1, representing the 2-fold difference, was added to the Ct values of the primary primers (*ache* S for wild-type and *ache* N for mutant) for homozygous while for heterozygotes the mean values of both primers were used.

For the AluYb8 primers as Walker *et al*.^38^ stated there could be two peaks in the melting curve analysis. We have confirmed that there was only one amplicon with the expected length (226 bp) upon running randomly selected qPCR products on the agarose gel although double Tm values were present in the qPCR results. Samples containing only the dimer were determined based on Tm analysis and excluded.

### Cloning, probe preparation and whole-mount *in situ* hybridization

Zebrafish *ache* coding sequence specific PCR fragment was amplified and T-A cloned into pGEMT-easy vector (Promega, Madison, USA). Plasmids were linearized by restriction enzyme digestion, purified and digoxygenin-labeled anti-sense RNA was produced using MAXIscript SP6/T7 *in vitro* transcription kit (Invitrogen, USA). Eluted probe was purified using LiCl method. *ache* whole-mount *in situ* hybridization (WISH) was performed using standard protocols^64^.

### AChE enzymatic activity assay

For measuring AChE/BChE enzymatic activity, an Ellman method based protocol was used as described by the manufacturer (Abnova, Taiwan). This assay was not AChE specific but rather measured the activity of both AChE and BChE (Abnova, personal communication). Briefly, homogenates from 5 × 10^6^ liver cancer cells (grown on 96 mm dishes) or zebrafish embryos (8 embryos per sample in triplicates at 3 dpf, grown at 28 or 33 °C) were prepared by passing samples 20 times through a syringe needle and subsequent centrifugation and supernatant collection. Homogenates were pipetted in triplicates and O.D. 412 was measured on an ELISA reader (BioTek, USA). Data from three measurements per group were averaged and activity was calculated as stated in the manufacturer’s protocol (Abnova, Taiwan).

### Liver cancer cell line maintenance

Liver cancer cell lines Hep3B, HepG2, Huh7, PLC, SKHep1 and Snu387 were maintained as previously described^44^. Briefly, Hep3B, HepG2, Huh7, PLC and SKHep1 lines were cultured in low glucose DMEM while Snu387 line was cultured in RPMI, and in both cases supplemented with 10% fetal bovine serum, 100 U/mL penicillin-streptomycin and 0.1 mM non-essential amino acids. In addition, Huh7 cells stably overexpressing GFP were supplemented with 200 μg/mL geneticin G-418 sulfate (Thermo Fisher Scientific, USA) and no additional antibiotics^34^. Cells were grown at 37 °C in an incubator supplied with 5% CO_2_. Cells reaching confluency (every 3 to 4 days) were split into new plates containing fresh media.

### Xenografting into *ache*^*sb55*^ embryos and tumorigenesis comparison with siblings

Xenotransplantation was performed using Hep3B and SKHep1 cells as well as Huh7-GFP cells as previously described for other cell types^65,66^. Briefly, cells were grown on T-75 cell culture flasks to confluency, trypsinized, and then counted using a hemocytometer before collecting 3 × 10^6^ cells for each sample. Hep3B or SKHep1 cells were washed once with PBS and then incubated with fluorescent dyes, for DiO (final 200 mM) at 37 °C for 20 minutes or for DiI (final 25 μg/ml; for AluYb8 experiment) at 37 °C for 5 minutes and following at 4°C for 15 minutes. Subsequently, cells were washed once with FBS and twice with PBS. Finally, cells were dissolved in 50 µl PBS containing a final 0.025% phenol red. At 2 dpf embryos were dechorionated using Pronase (Sigma, USA) and anesthetized in 0.4 mg/ml Tricaine (Fluka, USA). Cell suspension in approximately 10 nl volume containing approximately 300 cells was injected per embryo using an automated injector (Eppendorf FemtoJet, Germany). After injection of cells, embryos were screened using a Leica MZ10F stereo microscope at 6 hpi. Fish with fluorescent signal at the injection site were kept for further analysis while fish having signal outside of injection area or showing no signal were removed from the analysis. At 3 dpf, embryos were screened for the presence of paralysis, separated according to phenotype (Supplementary Figure 3). Embryos were incubated at 33 °C for 3 days after injection (dpi) at which they were fixed overnight at 4 °C in 4% paraformaldehyde; washed three times with PBS and then mounted in 50% glycerol. Fluorescent and brightfield images were taken using a Leica MZ10F stereo microscope (Leica, Switzerland) and analyzed using ImageJ software (NIH)^67^.

Tumor size from each embryo measured in ImageJ using freehand polygonal selection tool and the area encircled obtained as pixels for each image at the same magnification (x3.2). Additionally, segmentation was performed in ImageJ using threshold function for automated measurement of tumor area. Averaged manual-automatic tumor area measurements were used to construct graphs. For yolk sac measurements, a new set of embryos at 3 dpf and 5 dpf were bred. For tumor measurements fluorescent images and for yolk sac measurements brightfield images were used. Yolk sac measurements were performed manually using freehand polygonal selection tool in ImageJ (Supplementary Information for details). For quantification of metastasis, larvae with local tumor and migrated cells out of yolk were analyzed. Metastatic cell numbers were counted and larvae with more than 5 cells beyond yolk were counted as positive for metastasis^33^.

### Immunofluorescent staining and confocal imaging

At 3 dpi (5 dpf) the larvae injected with Huh7-GFP cells, as described for Hep3B and SKHep1 cells, were fixed using 4% paraformaldehyde (PFA) for 1 hour at room temperature and further permeabilized with cold methanol for 1 hour at 4 °C. Then, larvae were washed three times with PBS-Tween (0.3%) and blocked in PBDT (PBS, DMSO, Tween) supplemented with 2% normal sheep serum for 30 mins at room temperature. Rabbit phospho-histone H3 (ser10) (PH3) (Cell Signaling #9701) and mouse anti-GFP antibody (Cell Signaling, #2955) diluted 1:400 in blocking buffer and incubated with larvae overnight at 4 °C. After six washes with PBDT for a total of 2 hours, larvae were incubated with both anti-rabbit FITC and anti-mouse Cy3 tagged secondary antibody, respectively for PH3 and GFP proteins (Jackson’s Immuno Research, 711-096-152 and 715-167-003, respectively) diluted 1:400 in blocking buffer. Following six PBDT washes for a total of 2 hours, cells were stained with DAPI and washed three times in PBS-T. Finally, larvae were mounted in glycerol and imaged using a Zeiss LSM 880 confocal microscope (Zeiss, Germany). For tumor-focused images 20× objective was used and z-stacks at 10 micron intervals were taken using ZEN software (Zeiss, Germany) for each channel. 4 to 5 consecutive z-stacks were projected using ImageJ software’s Z-project function. Since the green GFP signal/background was not completely washed away it coincided with the green PH3 signal/puncta. Therefore, only images from the blue (DAPI) and red (GFP antibody) channels were used for merging to report the distribution of tumor cells in larvae.

### Statistical analyses

For two sample comparisons, student’s T-test was used while for multi-sample comparisons. One-Way ANOVA followed by Tukey’s test was performed in R^68^. Effects of genotype, cell line and experimental sets on tumor size were analyzed by a Three-Way ANOVA in R. To determine the interaction of genotypes with ALU Ct values (for human tumor DNA quantification) student’s T-test, One-Way ANOVA followed by Tukey’s test or Two-Way ANOVA in Matlab was performed (where indicated).

### Data availability

The datasets generated and/or analysed during the current study are available from the corresponding author on reasonable request.

## Electronic supplementary material

Supplementary Information

Supplementary Video 1

Supplementary Video 2
