# Supplementary material for: Development of a novel zebrafish xenograft model in ache mutants using liver cancer cell lines
Source: Sci Rep. 2018 Jan 25;8:1570. doi: 10.1038/s41598-018-19817-w (PMC5785479; doi:10.1038/s41598-018-19817-w)
Supplement: Supplementary file 1 — Supplementary Information [file 41598_2018_19817_MOESM1_ESM.pdf]

Development of a novel zebrafish xenograft model in *ache* mutants using liver cancer cell lines

M. Ender Avci[1,2,*], Ayse Gokce Keskus[5], Seniye Targen[1], M. Efe Isilak[1,6], Mehmet Ozturk[1,2], Rengul Cetin Atalay[3], Michelle M. Adams[4,5,6], Ozlen Konu[1,5,6]*

1. Department of Molecular Biology and Genetics, Bilkent University, 06800 Ankara, Turkey.

2. İzmir International Biomedicine and Genome Institute (iBG-izmir), Dokuz Eylul University, 35340 Izmir, Turkey.

3. Medical Informatics Department, Graduate School of Informatics, Middle East Technical University, 06800 Ankara, Turkey

4. Department of Psychology, Bilkent University, 06800 Ankara, Turkey.

5. Interdisciplinary Program in Neuroscience, Bilkent University, 06800 Ankara, Turkey.

6. UNAM-Institute of Materials Science and Nanotechnology, Bilkent University, 06800 Ankara, Turkey.

* Correspondence and requests for materials should be addressed to M.E.A. (ender.avci@bilkent.edu.tr) or O.K. (konu@fen.bilkent.edu.tr)

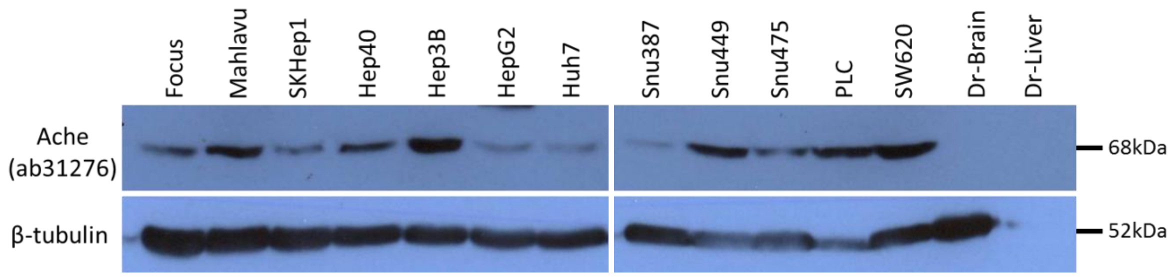

Supplementary Figure 1. Ache western blot in liver cancer cell lines and zebrafish brain, liver tissues. Liver cancer cell lines (and a colon carcinoma cell line SW620) were grown to confluency in 10 cm culture dishes in respective culture media and collected by scraping. Total protein was isolated using NP40 lysis buffer. Zebrafish liver and brain tissues dissected under stereo microscope and homogenates were prepared by passing through syringe 20 times in NP40 lysis buffer, followed by centrifugation and supernatant collection. Total protein amounts measured by Bradford assay and 50 µg protein was loaded in 5x loading buffer containing a final amount of 1% β-mercaptoethanol. Proteins were separated using 10% SDS-PAGE. Proteins were transferred to PVDF membrane by wet-transfer. Membrane was blocked overnight in 5% skim milk-TBST buffer at 4°C. Goat polyclonal Ache (Abcam-ab31276) antibody was diluted in above described blocking buffer at a final 1:400 concentration and membrane was incubated overnight at 4°C in this solution. After TBST washes, membrane was further incubated in secondary HRP conjugated goat polyclonal to rabbit IgG (H+L) at a final 1:5000 dilution in blocking buffer for 1 hr at room temperature. Following TBST washes, signal was developed using ECL+ (GE Healthcare) system. For equal loading, a rabbit polyclonal β-tubulin antibody was used. Figure comes from two separately run gels on the left and right handsights separated by a vertical solid white line, accordingly the same membranes were incubated with the above mentioned two different antibodies consequently following harsh membrane stripping.

Ache protein was detected in all studied HCC cell lines. There is low expression in HepG2, Huh7 and Snu387 cell lines. Ache antibody did not recognize zebrafish ache proteins due to lack of a respective band for zebrafish tissue homogenate sample wells.

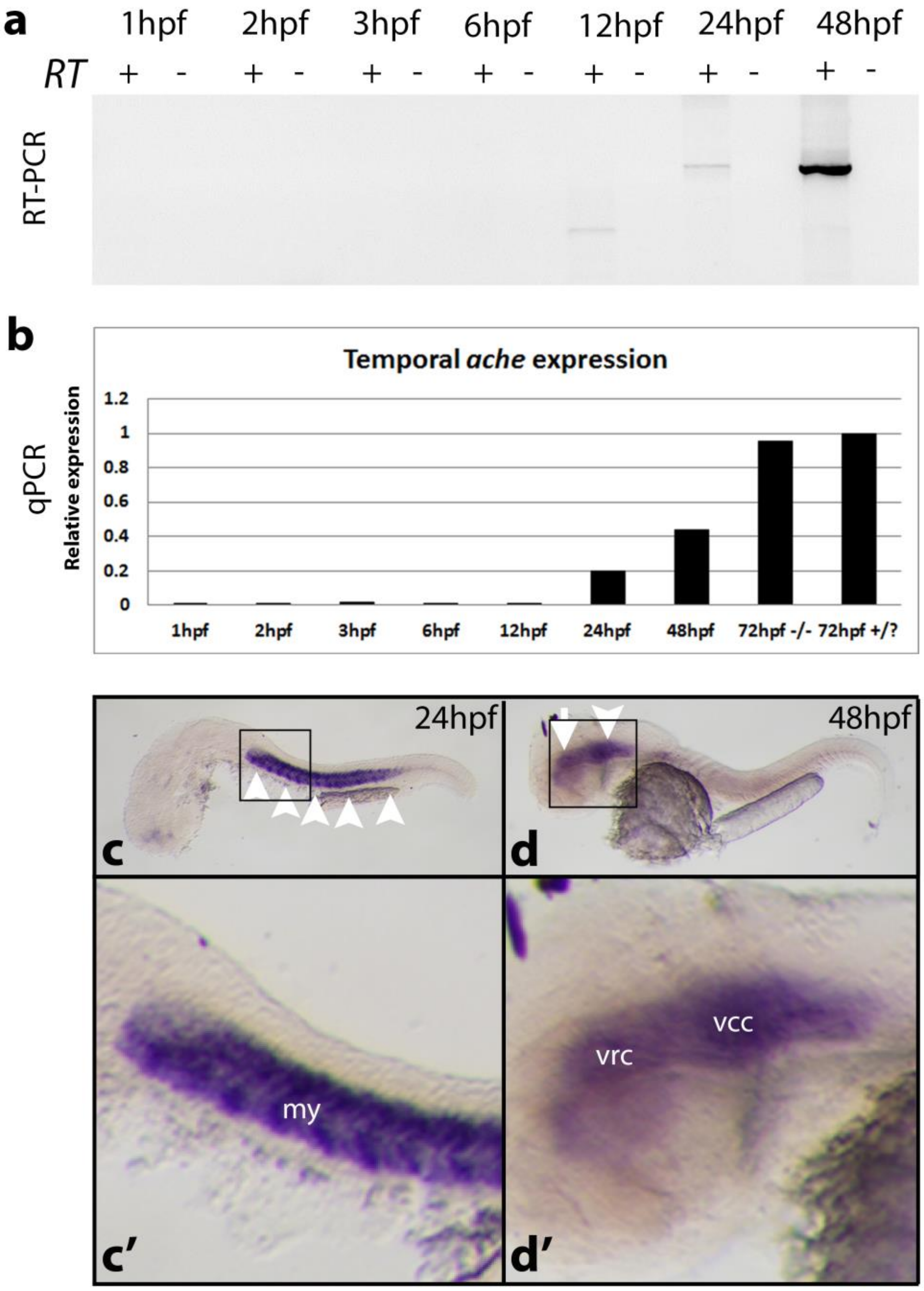

Supplementary Figure 2. *ache* expression analysis in AB line at different developmental stages. (**a**) *ache* expression was analyzed by using *ache* coding sequence spanning RT-PCR primers at 1, 2, 3, 6, 12, 24, and 48hpf. Amplification was performed for 35 cycles. cDNA was prepared from 1 microgram of total RNA. *ache* expression started between 12-24hpf. At 12 hpf there was a non-specific PCR band different than the expected amplicon size of 1905bp. (**b**) qPCR quantitative *ache* expression analysis with a new set of cDNA (N = 1 experiment; n = 20 embryos/group). In addition to the above stages RNA from *ache*-/- and *+/?* larvae at 72 hpf were included into analysis (N = 1 experiment; n = 20 embryos/group). At 72hpf *ache* expression was further increased compared to 48hpf expression level. *elfa* was used as housekeeping gene. *ache in situ* hybridization probes used to detect mRNA localization at (**c, c'**) 24 hpf where myotome (arrowheads) was stained; at (**d, d'**) 48 hpf where ventrorostral (arrow) and ventrocaudal clusters (arrowhead) in the inner brain were labeled. *my:* myotome, *vrc:* ventrorostral cluster, *vcc:* ventrocaudal cluster.

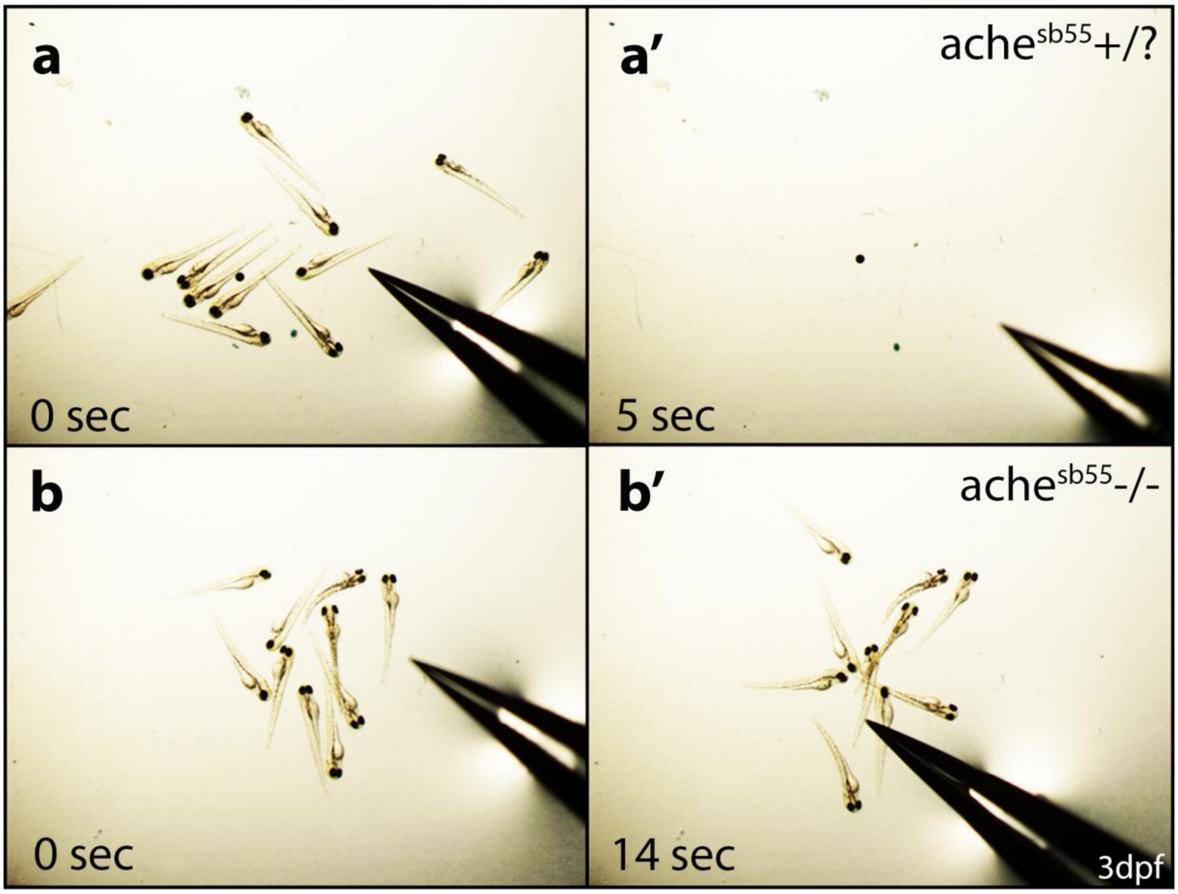

Supplementary Figure 3. *ache^{sb55}* mutant selection and characterization. Embryos obtained from *ache^{sb55}* heterozygote parental fish were grouped using "tail-test". (**a**) At 3 dpf, *ache* wild type siblings were able to swim following consecutive disturbance (**a'**). (**b**) Whereas, *ache^{sb55}* homozygous mutant fish were paralyzed due to excess ACh accumulation and could not move after touching their tails (**b'**).

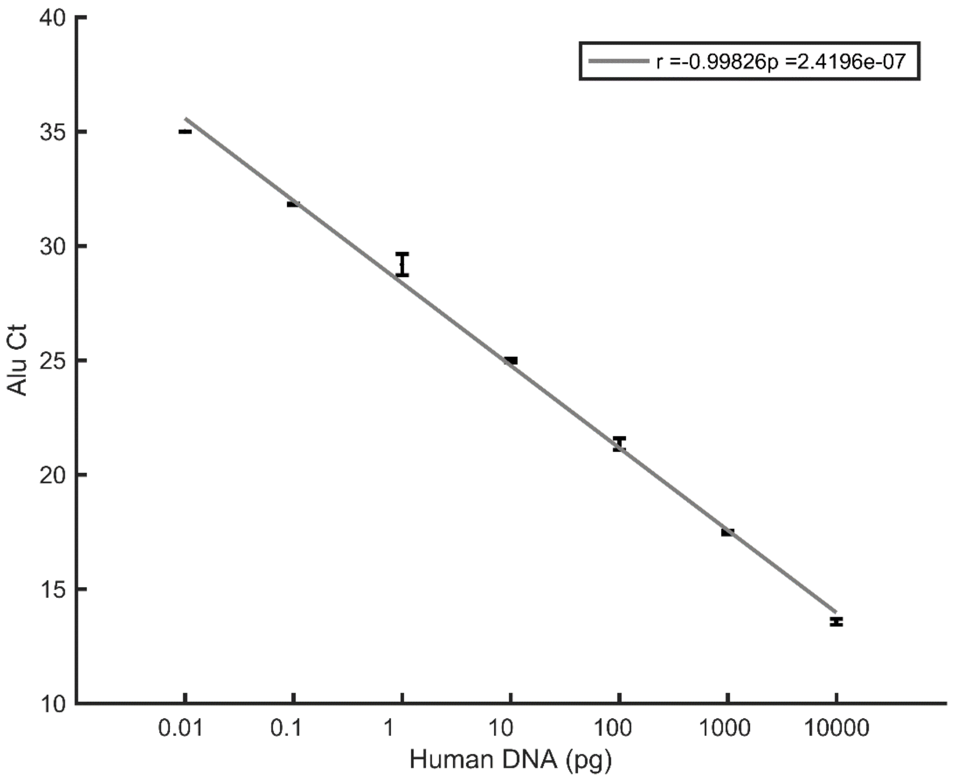

Supplementary Figure 4. Comparison of the human DNA amount (pg) across the AluYb8 Ct values. Serial dilutions from 10 ng to 0.01 pg of human DNA mixed with 10 ng of wild type zebrafish DNA were used for detection of AluYb8 using qPCR.

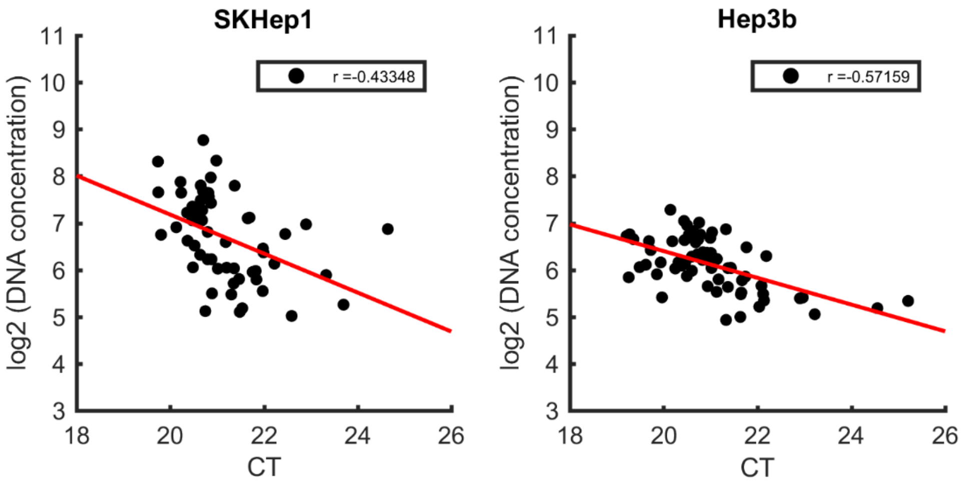

Suplementary Figure 5. The linear regression analysis of the zebrafish DNA amount measured with *ache* primers from embryos injected with Hep3B or SKHep1. Ct values of the main primer (added to 1) was used for the homozygous *ache+/+* or *ache-/-* larvae while mean of both primers was used for the heterozygous larvae (r = -0.43 and *P* = 9.46e-04 n=55 for SKHep1; r = -0.57 and *P* = 5.37e-07 n= 66 for Hep3B).

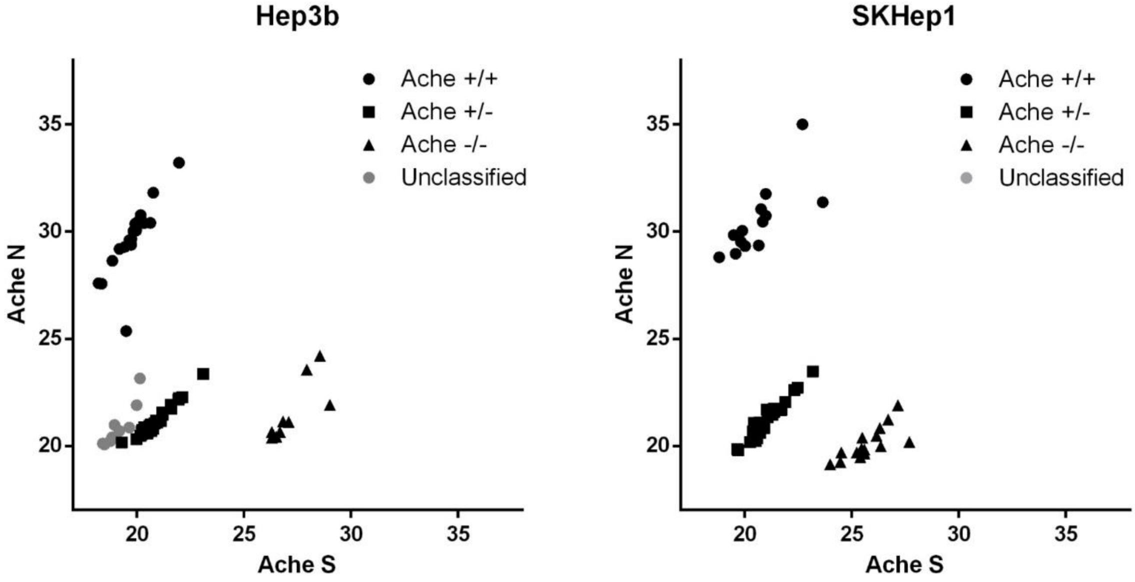

Supplementary Figure 6. Scatter plots of primer Ct values for genotyping of larvae injected with Hep3b or SKHep1. We considered the samples with an absolute Ct difference 1-4 cycles between primer sets as unclassified (shown with grey). The correlation coefficient between the Ct values of each allele was highly significant. For homozygous wild type larvae, the mean Ct difference between N-S primer set was 9.85 (±1.07; SD) and 9.85 (±1.12; SD) while for homozygous mutant larvae the mean Ct difference between *Ache* N – *Ache* S primer set was -5.65(±0.64; SD) and -5.7 (±0.8; SD) for SKHep1 and Hep3B, respectively.

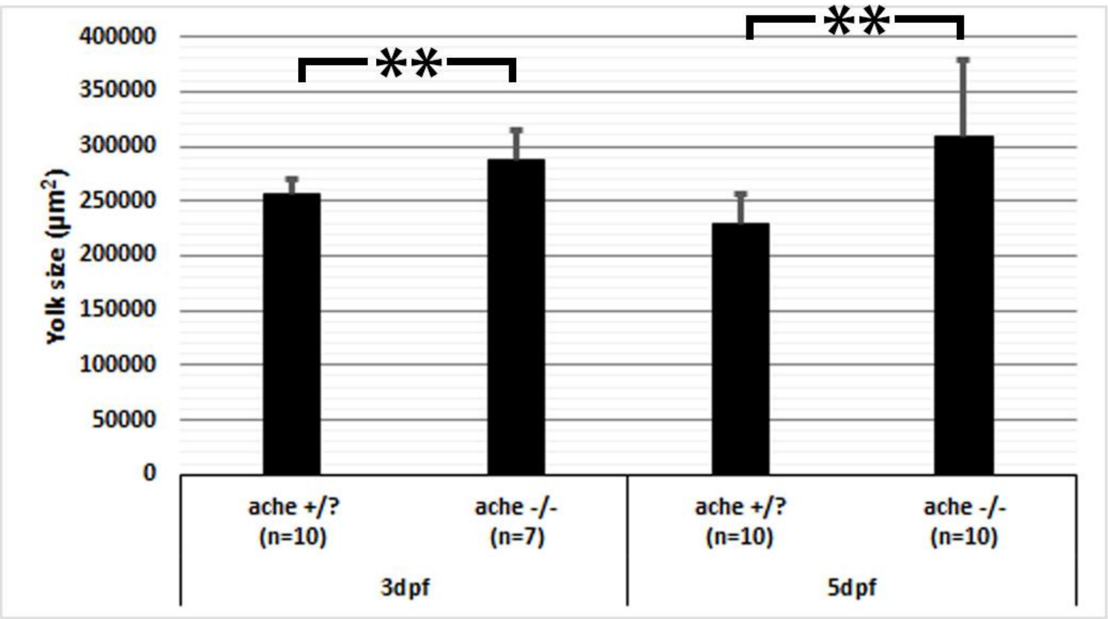

Supplementary Figure 7. Yolk sac size comparison between uninjected ache wild-type and mutant embryos at 3 and 5 dpf. 3 - 5 dpf embryos were anesthetized in Tricaine and imaged under brightfield. Yolk sizes were measured in ImageJ using freehand tool and then averaged for each group (3dpf - *ache+/?*, n = 10; 3dpf - *ache-/-*, n = 7; 5dpf - *ache+/?*, n = 10; 5dpf - *ache-/-*, n = 10). At 3dpf *ache* mutant embryos have 11% and at 5dpf 25% larger yolk size compared to wildtype siblings. ** $P < 0.01$.

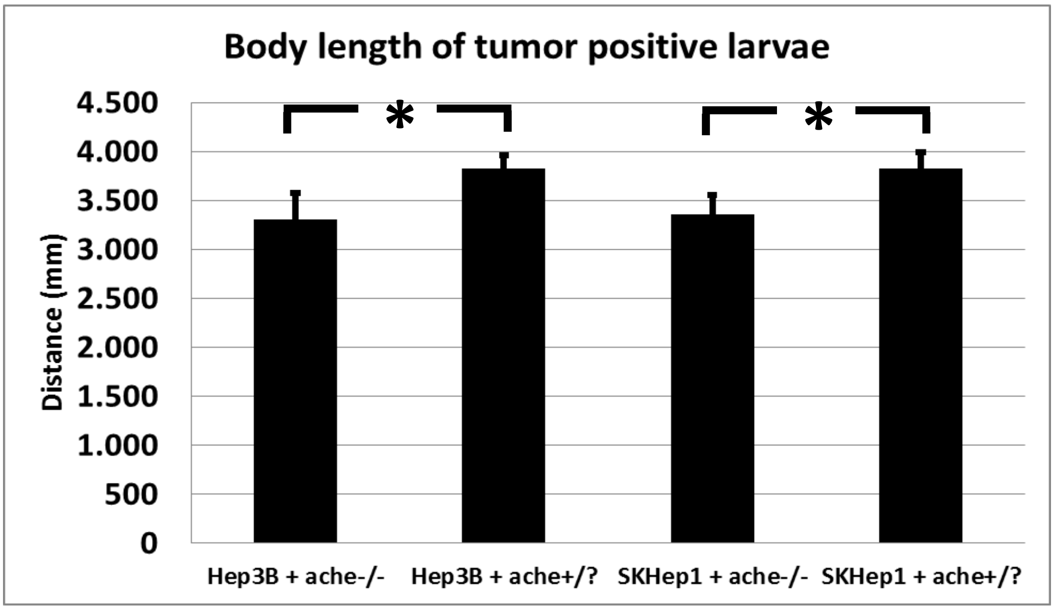

Supplementary Figure 8. Length measurements of *ache+/?* and *ache-/-* larvae with tumor mass at 5 dpf. Development of *ache* wild-type and mutant larvae were compared by measuring body lengths. To this end, head to tail (from tip to tip) distances of each larva was measured in ImageJ and then graphed. As shown in the graph, *ache-/-* larvae were on average 0.5 mm shorter in length when compared to *ache+/?* larvae. n = 26 (Hep3B+ *ache-/-*), n = 89 (Hep3B+ *ache+/?*), n = 20 (SKHep1+ *ache-/-*), n = 54 (SKHep1+ *ache+/?*). * $P < 0.05$.

Supplementary Table 1. Gene-specific primer sequences used in this study and their intended applications.

Gene ID	Forward (5’-3’)	Reverse (5’-3’)	Application
Hs_ACHEvar4-5	CAGCGACTGATGCGATACTG	GTGAAGCCTGGGCAGGTG	Q-PCR
Hs_ACHEvar4-6	GGGGCTCAGCAGTACGTTAG	TGCTGTAGTGGTCGAACTGG	Q-PCR
Hs_ACHEex3-4	TCTCGAAACTACACGGCAGA	CGCAGGTCCAGACTAACGTA	Q-PCR
Hs_BCHE	AGAATGGATGGGAGTGATGC	AGGCCAGCTTGTGCTATTGT	Q-PCR
Hs_TPT1	GATCGCGGACGGGTTGT	TTCAGCGGAGGCATTTCC	Q-PCR
Dr_ache	CACAGTTCTGTGCTCTGTGGA	TTCCACTGTCGCTCAACATC	Q-PCR
Dr_actb2	GCCTGACGGACAGGTCAT	ACCGCAAGATTCCATACCC	Q-PCR
Dr_elfa	CCCTGGACACAGAGACTTCA	CAGCCTCAAACTCACCAACA	Q-PCR
Dr_ache_CDS	ATGAAGACCTCAGATATTTTGCTCTTGCC	CTAGAGATCTGTGCAGCGCT CC	ISH probe preparation, RT-PCR
Dr_acheS	ACACGTGCCATATTGCAGAG	CTGCTCCAGGGAAGAACTTG	Genotyping
Dr_acheN	ACACGTGCCATATTGCAGAA	CTGCTCCAGGGAAGAACTTG	Genotyping
Hs_AluYb8	CGAGGCGGGTGGATCATGAGGT	TCTGTCGCCCAGGCCGGACT	Human DNA quantification
